# Plasma-Derived Exosomal microRNA-130a Serves as a Noninvasive Biomarker for Diagnosis and Prognosis of Oral Squamous Cell Carcinoma

**DOI:** 10.1155/2021/5547911

**Published:** 2021-04-16

**Authors:** Tao He, Xiangyu Guo, Xue Li, Chunjuan Liao, Xiaorong Wang, Kun He

**Affiliations:** State Key Laboratory of Oral Diseases & National Clinical Research Center for Oral Diseases, Department of Oral and Maxillofacial Surgery, West China Hospital of Stomatology, Sichuan University, Chengdu 610000, China

## Abstract

Exosomal microRNAs (miRNAs) are considered as potential stable biomarkers in many types of human cancer, but investigations of plasma-derived exosomal miRNAs in oral squamous cell carcinoma (OSCC) are still lacking. The aim of this study is to evaluate the diagnostic and prognostic values of exosomal miR-130a in OSCC patients. Exosomes were isolated from plasma samples which were collected from 184 OSCC patients before surgery and 196 healthy individuals. Primary OSCC and paired adjacent noncancerous tissues were also obtained from 47 OSCC patients. The expression levels of miR-130a were analyzed by quantitative real-time PCR (qRT-PCR). Our results showed that the expression levels of exosomal miR-130a were significantly higher in OSCC patients than those of the healthy controls (*p* < 0.0001). Also, the expression of miR-130a was also significantly upregulated in OSCC tissues compared with paired adjacent noncancerous tissues (*p* < 0.0001). A significant positive correlation was found between exosomal miR-130a and tissue miR-130a levels. Receiver operating characteristic (ROC) analyses yielded an AUC value of 0.812 in discriminating OSCC patients from healthy controls. Furthermore, high levels of exosomal miR-130a were associated with the late T-stage (*p*=0.024), advanced TNM stage (*p*=0.003), and poorly differentiated OSCC (*p*=0.013). Patients with high exosomal miR-130a expression had significantly worse 3-year overall survival (OS) and recurrence-free survival (RFS). Multivariate analysis indicated that exosomal miR-130a was an independent prognostic factor for OS (*p*=0.001) and RFS (*p*=0.003). Our results suggest that exosomal miR-130a may serve as a promising diagnostic and prognostic biomarker for OSCC patients.

## 1. Introduction

According to the latest cancer statistics, oral cancer is the leading cause of cancer-related morbidity among men, with high frequency in certain regions or specific countries, such as India and Sri Lanka [1]. In China, it is estimated that there are approximately 48,100 new cases diagnosed as oral cancer and 22,100 patients died of this malignancy in 2015 [2]. Over 90% of all oral cancers are classified as oral squamous cell carcinoma (OSCC) [3]. Although the therapeutic strategies have been greatly improved, the 5-year overall survival rate of OSCC patients is still less than 50%, which is mainly because of the high rates of the local recurrence or distant metastasis after primary treatment [4]. A histological examination on tissue biopsy of suspicious lesions is considered as the gold standard for OSCC diagnosis, but it is invasive, costly, and potentially harmful. Thus, most of OSCC cases are detected at advanced stages, which could lead to poor prognosis [3, 5]. Therefore, a novel diagnostic or prognostic biomarker which is less invasive and cost effective is urgently needed in clinical practice, and this could definitely improve patient survival.

Exosomes are nanometer-sized (30–150 nm) vesicles which are secreted by multivesicular bodies (MVBs). The main components of exosomes' external part are lipids, while various molecules such as proteins, mRNAs, microRNAs (miRNAs), genomic DNA, and mitochondrial DNA (mtDNA) are found inside [6, 7]. Tumor cells are known to secrete large amounts of exosomes which are at least 10-fold more than that of the normal cells and widely present in various body fluids, such as urine, saliva, blood, lymph, and cerebrospinal fluid [8, 9]. Exosomes could act as a delivery system which is important for communication between cells [10]. Therefore, exosomes are considered as an important component of the tumor microenvironment, which may contribute to tumor invasion and metastasis through communication with the surrounding stromal tissue or distant organs [11, 12]. MicroRNAs (miRNAs) are short (18–25 nucleotides long), single-stranded, noncoding RNAs which can regulate gene expression through inhibition of translation or degradation of the targeted mRNA [13]. As we all know, miRNAs have been proved to play critical roles in multiple cellular process of cancer, such as differentiation, proliferation, apoptosis, migration, and invasion [14]. Exosomes could envelop specific miRNAs and maintain its integrity in circulation, and exosomal miRNAs could possibly interfere with tumor microenvironment and facilitate tumor growth, invasion, and metastasis through taken up by surrounding or distant cells [15, 16]. Therefore, circulating exosomal miRNAs hold great promise to be used as novel noninvasive biomarkers in cancer diagnosis and prognosis.

Recently, accumulating evidences have confirmed that exosomal miRNAs are potential biomarkers for cancer diagnosis and prognosis, including lung cancer [17], liver cancer [18], colorectal cancer [19], and breast cancer [20]. In OSCC, only few studies have explored the association between the exosomal miRNAs and tumor growth, invasion, and metastasis, such as miR-24-3p and miR-382-5p [21, 22]. As a member of the microRNA-130 family, miR-130a has been extensively studied in various types of human cancer. However, the results are inconsistent and disputable. Several studies have demonstrated that miR-130a serves as a tumor suppressor in liver cancer and prostate cancer [23, 24], while miR-130a has also been reported to exhibit tumorigenic characteristics in breast cancer, esophageal cancer and cervical cancer [25–27]. Moreover, the diagnostic and prognostic value of miR-130a in OSCC is still unknown. In the present study, we aim to investigate the correlation between plasma-derived exosomal miR-130a and clinicopathological characteristics of OSCC and the potential of using exosomal miR-130a as a biomarker for diagnosis and prognosis in OSCC patients.

## 2. Material and Methods

### 2.1. Clinical Samples

The present study was approved by the ethics committee of West China Hospital. A total of 184 patients with OSCC who underwent surgical resection at our hospital and 196 age and sex matched healthy controls were recruited between 2014 and 2016. None of the OSCC patients received radiotherapy or chemotherapy before surgery. All tumors were staged according to the seventh edition of the American Joint Committee on Cancer system. Blood samples were collected in EDTA tubes from all OSCC patients before surgery and healthy individuals. For plasma, fresh blood was centrifuged at 2000g for 10 min, and then, the supernatant was followed by a second centrifugation at 5000g for 15 min at 4°C. In addition, primary OSCC and paired adjacent noncancerous tissues were also obtained from 47 out of 184 patients. All samples were stored at −80°C until analysis. Written informed consent was obtained from all participants. Clinicopathological information including age, gender, and TNM stage was collected by reviewing the medical records. All patients were followed up for 3 years to record survival conditions. The median follow-up period was 31 months (range, 5–36 months).

### 2.2. Isolation of Exosomes from Plasma

Exosomes were extracted from plasma using the ExoQuick ULTRA EV Isolation Kit for Serum and Plasma (SBI System Biosciences, US) according to the manufacturer's protocol. Briefly, ExoQuick ULTRA was added to 250 *µ*L plasma and incubated on ice for 30 min; after that, the mixture was centrifuged at 3000g for 10 min. Extracellular vesicles (EVs) in the pellet were resuspended and added to prewashed ExoQuick ULTRA columns, and then, the purified exosomes were obtained after a second centrifugation at 1000g for 30 s.

### 2.3. Quantification of Exosomal miRNAs

Total RNA of exosomes was extracted using the miRNeasy Serum/Plasma Kit (Qiagen, Germany) according to the manufacturer instructions, while total RNA of tumor tissues and paired adjacent noncancerous tissues were extracted using the miRNeasy Mini Kit (Qiagen, Germany). RNA quality was assessed using a NanoDrop^™^ 8000 spectrophotometer (Thermo Scientific, US). Complementary DNA (cDNA) was synthesized using TaqMan MicroRNA primers specific for miR-130a and a TaqMan MicroRNA Reverse-Transcription Kit (Thermo Scientific, US). Quantitative reverse-transcription polymerase chain reaction (qRT-PCR) was performed in triplicate on the Bio-Rad CFX Connect Real-Time PCR Detection System (Bio-rad, US). The PCR conditions were 95°C for 10 min, followed by 40 cycles of 95°C for 15 s and 60°C for 1 min. In addition, a synthetic Caenorhabditis elegans miR-39 RNA oligonucleotide (cel-miR-39 mimic) was used as a spike-in control in plasma exosomes, while U6 small nuclear RNA was used as an internal control in tissues. Relative quantification of miR-130a expression was calculated using the 2^−ΔΔCT^ method.

### 2.4. Statistical Analysis

Statistical analyses were performed using the SPSS statistical software package (version 19.0, SPSS lnc., US) and GraphPad Prism (version 5.0, GraphPad Software Inc., US). Student's *t*-test was used to determine statistically significant differences between groups. The association between miR-130a expression and clinicopathological characteristics was analyzed by the *χ*^2^ test. Receiver operating characteristic (ROC) curves were established to evaluate the diagnostic value of miR-130a, and the area under the curve (AUC) with 95% confidence interval (CI) was also calculated. Overall survival (OS) and recurrence-free survival (RFS) curves were constructed using the Kaplan–Meier method, and the differences were examined using log-rank tests. Univariate and multivariate hazard ratios for OS and RFS were estimated using a Cox proportional hazards regression model. A two-tailed *p* value < 0.05 was considered statistically significant.

## 3. Results

### 3.1. Characteristics of the Study Population

The clinical characteristics of the 184 OSCC patients and 196 healthy volunteers are summarized in Table 1. The control individuals were frequently matched with patients by age and gender. The mean ages of the patients and the controls were 56.3 ± 16.4 and 55.5 ± 16.6 years, respectively (*p*=0.947). No significantly statistical differences were noted in the distributions of the smoking habit (*p*=0.12) and drinking habit (*p*=0.475) between the patients and controls. Most of the patients had early T-stage, negative N-stage, and well-differentiated or moderately differentiated OSCC. Eighty patients were diagnosed at advanced TNM stage. Ninety patients had experienced tumor recurrence after surgical resection, and 63 patients had died at the end of 3-year follow-up. The median RFS was 28.9 months (Supplementary File 1).

### 3.2. Expression of miR-130a in OSCC Tissues and Plasma Exosomes

**Figure 1:**
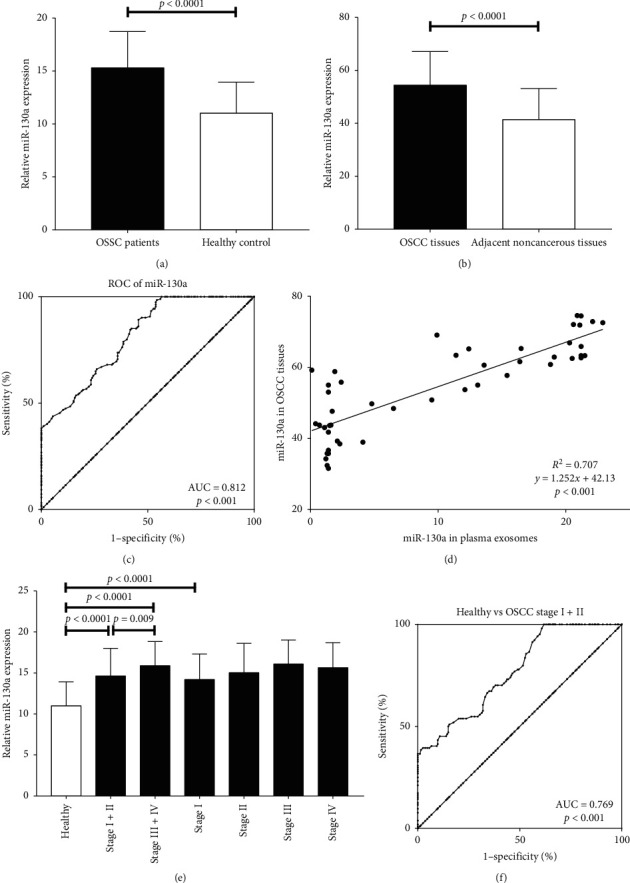
miR-130a expression is significantly higher in OSCC patients. (a) Comparison of exosomal miR-130a expression levels between OSCC patients and healthy controls. (b) Comparison of miR-130a expression levels between OSCC tissues and paired adjacent noncancerous tissues. (c) ROC curves of exosomal miR-130a for discriminating OSCC patients from healthy controls. (d) Correlation between expression of miR-130a in plasma exosomes and that in OSCC tissues. (e) Comparison of miR-130a expression levels between OSCC patients at different TNM stages and healthy controls. (f) ROC curves of exosomal miR-130a for discriminating patients with early-stage OSCC from healthy controls.

Expression of miR-130a was assessed by qRT-PCR. As shown in Figure 1(a), the results showed that the relative expression levels of exosomal miR-130a were significantly higher in OSCC patients than those of the healthy controls (*p* < 0.0001). For OSCC patients, the expression of miR-130a was significantly upregulated in OSCC tissues (54.5 ± 12.6, mean relative expression level ± SD) when compared with that in paired adjacent noncancerous tissues (41.5 ± 11.6, Figure 1(b)). Furthermore, the correlation between exosomal miR-130a levels and miR-130a expression in OSCC tissues in the same patients were examined, and a significant positive correlation was demonstrated between them (*p* < 0.001, Figure 1(d)). We also compared the expression levels of exosomal miR-130a in OSCC patients with different TNM stages, and miR-130a levels were still significantly lower in healthy controls than OSCC patients with TNM stage I (*p* < 0.001), as well as TNM stage II, III, and IV (Figure 1(e) and Table 2). In addition, the results also showed that miR-130a levels were significantly higher in stage III and IV compared to stage I and II (*p*=0.009, Table 2).

### 3.3. Exosomal miR-130a as Diagnosis Signature for OSCC

ROC curve analysis was performed to know whether exosomal miR-130a could sensitively and specifically discriminate the OSCC patients from the healthy controls. The area under the curve (AUC) value of exosomal miR-130a was 0.812 (95% confidence interval (CI) = 0.77–0.853, Figure 1(c)). An optimal cut-off value was indicated at 15.85 with a sensitivity of 98.5% and a specificity of 45.7%. Furthermore, the AUC value obtained for exosomal miR-130a to distinguish the OSCC patients in the early stage (I and II) from the healthy controls was 0.769 (95% CI = 0.715–0.823, Figure 1(f)), and an optimal cut-off value was indicated at 10.05 with a sensitivity of 100% and a specificity of 38.1%. These results indicated that exosomal miR-130a might have a potential diagnostic value for distinguishing the OSCC patients from the healthy controls.

### 3.4. Clinicopathological Significance of Exosomal miR-130a Expression

For clinicopathological analysis, patients were classified into high- and low-expression groups using the median relative expression level of exosomal miR-130a. Our results revealed that high expression of exosomal miR-130a was significantly correlated with the late T-stage (*p*=0.024), advanced TNM-stage (*p*=0.003), and poorly differentiated OSCC (*p*=0.013). However, there were no significant correlations of exosomal miR-130a expression with other clinical variables such as the age, gender, smoking and drinking habit, tumor site, and *N*-stage (Table 3).

### 3.5. Correlation between Exosomal miR-130a Expression and Prognosis of OSCC Patients

**Figure 2:**
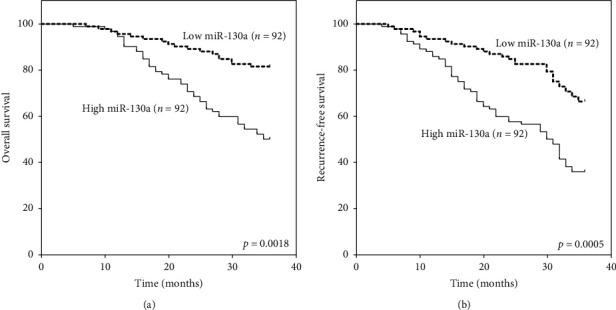
Survival analysis of 184 patients with OSCC by the Kaplan–Meier method. Overall survival and recurrence-free survival of patients with high and low expression of plasma-derived exosomal miR-130a. *p* values were calculated using a two-sided log-rank test.

To evaluate the prognostic significance of exosomal miR-130a expression, the Kaplan–Meier OS and RFS curves of the 184 OSCC patients were compared between those with high and those with low levels of exosomal miR-130a. The results showed that OSCC patients with high exosomal miR-130a expression had shorter 3-year OS (*p*=0.0018, Figure 2(a)) and RFS (*p*=0.0005, Figure 2(b)) than those with low exosomal miR-130a expression. Univariate analysis showed that exosomal miR-130a expression levels, T-stage, TNM stage, and histologic grade were significantly correlated with OS and RFS (*p* < 0.05), whereas age, gender, smoking and drinking habit, tumor site, or N-stage were not (Tables 4 and 5). Multivariate Cox regression analysis was performed for those factors which showed significance in univariate analysis. Also, our results showed that exosomal miR-130a expression levels (*p*=0.001), TNM stage (*p*=0.037), and histologic grade (*p*=0.025) were independent prognostic factors for predicting the 3-year OS of OSCC patients (Table 4). Furthermore, in RFS, multivariate analysis demonstrated that exosomal miR-130a expression levels (*p*=0.003), T-stage (*p*=0.028), TNM stage (*p*=0.03), and histologic grade (*p*=0.043) emerged as significant independent prognostic factors (Table 5).

## 4. Discussion

In recent years, liquid biopsy has received much more attention as a novel noninvasive diagnostic or prognostic tool for providing real-time information about cancer, which was based on the detection of circulating tumour DNA (ctDNA), circulating tumour cells (CTCs), exosomal miRNAs, and so on [28]. Analyses of these CTCs or circulating nucleic acids could be applied to cancer early diagnosis, response monitoring, and drug resistance assessment [28]. The key advantages of liquid biopsy are that it could provide greater accuracy of tumour heterogeneity than tissue-based biopsy and collect samples in a minimally invasive way. Particularly, exosomal miRNAs have been proved to be existed not only in blood but also in other multiple body fluids, such as urine, saliva, pleural effusions, and cerebrospinal fluid [9]. Up to now, at least 50 exosomal miRNAs have been proved to play a role in cancer diagnosis and prognosis [29, 30]. However, to the best of our knowledge, there is only one study by He et al. which demonstrated that salivary exosomal miR-24-3p could be used as a candidate screening biomarker for OSCC and it can enhance the proliferative capacity of OSCC cells through targeting PER 1 [21]. In the present study, we provided the first investigation in the diagnostic and prognostic value of plasma-derived exosomal miR-130a expression in OSCC.

The previous study by Hilly et al. provided the first evidence that miR-130a expression in OSCC tissues from young patients with recurrence was upregulated as compared with the patients without recurrence [31]. In a recent study by Yang et al., it was also reported that miR-130a was highly expressed in OSCC tissues and significantly associated with the TNM stage and lymph node metastasis [32]. Consistent with that, in our study, we found that the expression of miR-130a was upregulated in OSCC tissues when compared with that in paired adjacent noncancerous tissues, and it was significantly associated with more aggressive clinicopathological characteristics of OSCC patients, including late T-stage, advanced TNM stage, and poorly differentiated grade. Previous studies have reported that miR-130a could facilitate proliferation and metastasis of tumor cells through downregulating the expression of tumor suppressor genes, such as phosphatase and tensin homolog (PTEN) in osteosarcoma and breast cancer [25, 33], peroxisome proliferator-activated receptor gamma (PPARG) in chorangiocarcinoma [34], and collapsing-response mediator protein type 4 (CRMP4) in gastric cancer [35]. In two oral tongue squamous cell carcinoma (OTSCC) cell lines CAL-27 and SSC-4, cylindromatosis (CYLD), which acts as a tumor suppressor in several malignancies and is responsible for cisplatin resistance in OSCC patients [36, 37], has been proved to be one of the multiple target genes of miR-130a [32]. These are supposed to be the mechanisms of its correlation with more aggressive tumors. Further studies are needed. Moreover, our results also demonstrated that the expression of plasma-derived exosomal miR-130a was significantly higher in OSCC patients than that of the healthy volunteers. Also, there was a significant positive correlation between expression of miR-130a in plasma exosomes and primary OSCC tissues, suggesting that tumor tissue may be the source of circulating exosomal miRNAs. ROC curve analysis revealed that exosomal miR-130a holds great promise to be used as a novel diagnostic biomarker for the screening of OSCC.

Accumulating evidence has suggested that miR-130a could be used as potential prognostic biomarkers for certain types of cancer. Fox example, a study by Jiang H et al. reported that high expression of miR-130a promoted gastric cancer tumorigenesis by targeting runt-related transcription factor 3 (RUNX3) and was significantly associated with poor OS [38]. Contrastingly, Kaplan–Meier analysis by Li B et al. demonstrated that high expression of miR-130a in patients with hepatocellular carcinoma (HCC) had significantly improved OS [23]. In a recent meta-analysis, Peng et al. evaluated 12 eligible studies comprising 1582 cases with various types of cancer, including HCC, colorectal cancer, gastric cancer, lymphoma, non-small-cell lung cancer, cervical cancer, cholangiocarcinoma, and osteosarcoma, and the pooled hazard ratios (HRs) indicated a significant relationship between the high levels of miR-130a expression and poor OS in cancer patients (HR = 1.58, 95%CI = 1.21–2.06, *p* < 0.001) [39]. Up to now, the prognostic role of miR-130a has not been investigated in OSCC. In the present study, our results showed that OSCC patients with high exosomal miR-130a expression had a significantly worse 3-year OS and RFS than those with low exosomal miR-130a expression. Also, multivariate analysis further confirmed that miR-130a was a prognostic biomarker independent of several adjusted well-known prognostic parameters for OSCC, including T-stage, TNM stage, and histologic grade. However, the small sample size, the retrospective nature of this study, and the lack of underlying biological mechanisms for the effects of miR-130a on the occurrence, development, and prognosis of OSCC did not allow us to draw any concrete conclusions yet. Further studies are required to confirm the present findings. Moreover, it is probably meaningful to monitor the changes of exosomal miR-130a levels after surgery because it might be associated with tumor recurrence, side effects of treatment, survival quality, and so on, and further studies are also required.

## 5. Conclusions

In conclusion, our results provided the first evidence that plasma-derived exosomal miR-130a expression was significantly increased in OSCC patients and associated with late T-stage, advanced TNM stage, and poorly differentiated grade. Moreover, exosomal miR-130a may serve as a promising diagnostic and prognostic biomarker for OSCC patients.

## Figures and Tables

**Table 1:** Clinical characteristics of participants.

Variable	Patient (*N* = 184)	Control (*N* = 196)	*p* value
*Age (years, mean* *±* *SD)*	56.3 ± 16.4	55.5 ± 16.6	0.947

*Age group (years), N (%)*			
≤60	82 (44.6)	87 (44.4)	0.972
> 60	102 (55.4)	109 (55.6)	

*Gender, N (%)*			
Male	125 (67.9)	134 (68.4)	0.928
Female	59 (32.1)	62 (31.6)	

*Smoking habit, N (%)*			
Yes	102 (55.4)	93 (47.4)	0.12
No	82 (44.6)	103 (52.6)	

*Drinking habit, N (%)*			
Yes	134 (72.8)	149 (76)	0.475
No	50 (27.2)	47 (24)	

*Tumor site, N (%)*			
Tongue	83 (45.1)		
Nontongue	101 (54.9)		

*T-stage, N (%)*			
T1 + T2	111 (60.3)		
T3 + T4	73 (39.7)		

*N-stage, N (%)*			
Positive	69 (37.5)		
Negative	115 (62.5)		

*TNM stage, N (%)*			
I-II	104 (56.5)		
III-IV	80 (43.5)		

*Histologic grade, N (%)*			
Well + moderate	120 (65.2)		
Poor	64 (34.8)		

^*∗*^Mean *p* value<0.05.

**Table 2:** Performance of exosomal miR-130a for detection of OSCC with different TNM stages.

Group	Healthy control	Stage I + II	Stage III + IV	Stage I	Stage II	Stage III	Stage IV
Healthy control	—	—	—	—	—	—	—
Stage I + II	<0.0001^*∗*^	—	—	—	—	—	—
Stage III + IV	<0.0001^*∗*^	0.009^*∗*^	—	—	—	—	—
Stage I	<0.0001^*∗*^	0.5	0.002^*∗*^	—	—	—	—
Stage II	<0.0001^*∗*^	0.533	0.164	0.262	—	—	—
Stage III	<0.0001^*∗*^	0.015^*∗*^	0.706	0.005^*∗*^	0.133	—	—
Stage IV	<0.0001^*∗*^	0.084	0.687	0.02^*∗*^	0.417	0.501	—

^*∗*^Mean *p* value<0.05.

**Table 3:** Clinicopathological characteristics of patients with OSCC.

Clinicopathological factors	No.	Exosomal miR-130a expression		
		Low, N (%)	High, N (%)	*p* value
*Age*				
< 60 years	82	43 (23.4)	39 (21.2)	0.553
≥ 60 years	102	49 (26.6)	53 (28.8)	

*Gender*				
Male	125	59 (32.1)	66 (35.9)	0.269
Female	59	33 (17.9)	26 (14.1)	

*Smoking habit*				
Yes	102	50 (27.2)	52 (28.3)	0.767
No	82	42 (22.8)	40 (21.7)	

*Drinking habit*				
Yes	134	65 (35.3)	69 (37.5)	0.507
No	50	27 (14.7)	23 (12.5)	

*Tumor site*				
Tongue	83	43 (23.4)	40 (21.7)	0.657
Nontongue	101	49 (26.6)	52 (28.3)	

*T-stage*				
T1 + T2	111	63 (34.2)	48 (26.1)	0.024^*∗*^
T3 + T4	73	29 (15.8)	44 (23.9)	

*N-stage*				
Negative	115	63 (34.2)	52 (28.3)	0.094
Positive	69	29 (15.8)	40 (21.7)	

*TNM stage*				
I-II	104	62 (33.7)	42 (22.8)	0.003^*∗*^
III-IV	80	30 (16.3)	50 (27.2)	

*Histologic grade*				
Well + moderate	120	68 (37)	52 (28.3)	0.013^*∗*^
Poor	64	24 (13)	40 (21.7)	

^*∗*^Mean *p* value<0.05.

**Table 4:** Univariate and multivariate analyses of overall survival in 184 patients with OSCC.

Variable	Univariate analysis		Multivariate analysis	
	HR (95% CI)	*p* value	HR (95% CI)	*p* value
Exosomal miR-130a (high)	4.412 (2.265–8.591)	<0.001^*∗*^	3.276 (1.624–6.607)	0.001^*∗*^
Age (≥60 years)	1.007 (0.546–1.859)	0.981		
Gender (male)	1.358 (0.713–2.585)	0.352		
Smoking habit (yes)	1.206 (0.654–2.224)	0.548		
Drinking habit (yes)	1.482 (0.728–3.016)	0.277		
Tumor site (tongue)	1.039 (0.555–1.948)	0.904		
T-stage (T3 + T4)	2.227 (1.195–4.153)	0.012^*∗*^	1.853 (0.933–3.682)	0.078
N-stage (positive)	1.546 (0.831–2.877)	0.169		
TNM stage (III-IV)	2.581 (1.382–4.821)	0.003^*∗*^	2.07 (1.046–4.097)	0.037^*∗*^
Histologic grade (poor)	2.871 (1.517–5.434)	0.001^*∗*^	2.199 (1.102–4.389)	0.025^*∗*^

HR: hazard ratio; CI: confidence interval; ^*∗*^mean *p* value<0.05.

**Table 5:** Univariate and multivariate analyses of recurrence-free survival in 184 patients with OSCC.

Variable	Univariate analysis		Multivariate analysis	
	HR (95% CI)	*p* value	HR (95% CI)	*p* value
Exosomal miR-130a (high)	3.518 (1.917–6.455)	<0.001^*∗*^	2.627 (1.382–4.993)	0.003^*∗*^
Age (≥60 years)	1.181 (0.66–2.114)	0.575		
Gender (male)	1.121 (0.603–2.082)	0.718		
Smoking habit (yes)	1.41 (0.786–2.527)	0.249		
Drinking habit (yes)	1.051 (0.549–2.014)	0.88		
Tumor site (tongue)	1.072 (0.59–1.948)	0.819		
T-stage (T3 + T4)	2.357 (1.287–4.317)	0.005^*∗*^	2.086 (1.082–4.023)	0.028^*∗*^
N-stage (positive)	1.64 (0.913–2.946)	0.098		
TNM stage (III-IV)	2.429 (1.336–4.414)	0.004^*∗*^	2.056 (1.073–3.936)	0.03^*∗*^
Histologic grade (poor)	2.583 (1.38–4.836)	0.003^*∗*^	2.007 (1.023–3.938)	0.043^*∗*^

HR: hazard ratio; CI: confidence interval; ^*∗*^mean *p* value<0.05.

## Data Availability

The data used to support the findings of this study are available from the corresponding author upon request.

## Supplementary Materials

Supplementary Materials Table 1: clinical characteristics of patients. Table 2: clinical characteristics of controls.
