# Supplementary material for: Plasma-Derived Exosomal microRNA-130a Serves as a Noninvasive Biomarker for Diagnosis and Prognosis of Oral Squamous Cell Carcinoma
Source: J Oncol. 2021 Apr 16;2021:5547911. doi: 10.1155/2021/5547911 (PMC8068531; doi:10.1155/2021/5547911)
Supplement: Supplementary Materials — Table 1: clinical characteristics of patients. Table 2: clinical characteristics of controls. [file 5547911.f1.docx]

**Table 1.** **Clinical characteristics of patients**

| **Patient ID** | **gender** | **age** | **smoking** | **drinking** | **tumor site** | **TNM stage** | **Histologic grade** | **overall survival (month)** | **recurrence (month)** | **miR-130a value** |
| --- | --- | --- | --- | --- | --- | --- | --- | --- | --- | --- |
| P1 | Male | 66 | Yes | Yes | Tongue | III | poor | 36 | 36 | 16.4 |
| P2 | Male | 29 | No | Yes | Non-tongue | II | Well | 36 | 36 | 12.9 |
| P3 | Male | 46 | No | No | Tongue | II | poor | 36 | 33 | 20.2 |
| P4 | Female | 48 | No | No | Non-tongue | I | Well | 36 | 36 | 13 |
| P5 | Male | 62 | No | Yes | Non-tongue | I | Moderate | 31 | 26 | 17.3 |
| P6 | Female | 30 | Yes | No | Non-tongue | II | Well | 36 | 30 | 11.4 |
| P7 | Male | 57 | No | Yes | Tongue | I | poor | 36 | 36 | 10.2 |
| P8 | Male | 70 | **Yes** | Yes | Non-tongue | IV | poor | 11 | 8 | 20.7 |
| P9 | Female | 29 | No | Yes | Non-tongue | II | Well | 23 | 17 | 10.7 |
| P10 | Male | 71 | No | No | Tongue | III | Well | 36 | 32 | 18.9 |
| P11 | Male | 73 | **Yes** | No | Tongue | I | Well | 36 | 36 | 14.1 |
| P12 | Male | 68 | Yes | No | Non-tongue | IV | poor | 15 | 12 | 16.1 |
| P13 | Male | 67 | No | Yes | Tongue | I | Well | 36 | 36 | 10.6 |
| P14 | Male | 69 | **Yes** | Yes | Tongue | IV | poor | 36 | 34 | 11.9 |
| P15 | Male | 46 | Yes | Yes | Non-tongue | III | Moderate | 36 | 31 | 14.4 |
| P16 | Male | 71 | No | Yes | Tongue | I | Moderate | 36 | 36 | 15.1 |
| P17 | Male | 24 | Yes | Yes | Tongue | II | Moderate | 35 | 30 | 18.2 |
| P18 | Male | 67 | No | Yes | Non-tongue | II | Well | 28 | 24 | 15.9 |
| P19 | Female | 65 | No | Yes | Tongue | III | poor | 16 | 12 | 19.8 |
| P20 | Female | 50 | **Yes** | No | Non-tongue | III | Well | 36 | 36 | 12 |
| P21 | Female | 57 | No | Yes | Non-tongue | I | poor | 14 | 11 | 10.2 |
| P22 | Male | 32 | No | No | Tongue | I | Moderate | 36 | 36 | 10.8 |
| P23 | Male | 66 | **Yes** | Yes | Tongue | II | Moderate | 23 | 17 | 20.2 |
| P24 | Male | 25 | Yes | Yes | Non-tongue | II | Moderate | 25 | 22 | 19.6 |
| P25 | Male | 61 | Yes | Yes | Tongue | I | Moderate | 36 | 36 | 18.7 |
| P26 | Female | 75 | **Yes** | Yes | Non-tongue | IV | poor | 17 | 11 | 20.8 |
| P27 | Male | 50 | Yes | Yes | Non-tongue | IV | Moderate | 36 | 33 | 16.6 |
| P28 | Male | 68 | **Yes** | Yes | Tongue | IV | Moderate | 36 | 36 | 17.8 |
| P29 | Male | 32 | No | Yes | Non-tongue | I | Moderate | 36 | 36 | 13.3 |
| P30 | Male | 69 | Yes | Yes | Tongue | III | Well | 36 | 36 | 15.2 |
| P31 | Male | 68 | No | Yes | Non-tongue | II | poor | 36 | 36 | 17.7 |
| P32 | Male | 70 | Yes | Yes | Non-tongue | I | poor | 33 | 30 | 12.9 |
| P33 | Male | 69 | Yes | No | Non-tongue | I | Moderate | 36 | 36 | 16.8 |
| P34 | Male | 70 | Yes | Yes | Non-tongue | II | Moderate | 26 | 24 | 19.5 |
| P35 | Male | 65 | No | Yes | Non-tongue | I | poor | 36 | 36 | 14.4 |
| P36 | Male | 69 | Yes | Yes | Non-tongue | IV | Well | 36 | 30 | 16.5 |
| P37 | Male | 62 | No | Yes | Tongue | IV | Well | 11 | 9 | 14 |
| P38 | Male | 45 | No | No | Tongue | II | Moderate | 36 | 36 | 10.4 |
| P39 | Male | 59 | **No** | Yes | Tongue | II | poor | 36 | 36 | 18.3 |
| P40 | Male | 44 | Yes | Yes | Tongue | II | Moderate | 36 | 36 | 13.1 |
| P41 | Male | 20 | No | No | Tongue | I | Well | 31 | 20 | 20.8 |
| P42 | Female | 71 | Yes | Yes | Tongue | IV | Well | 36 | 36 | 13.6 |
| P43 | Male | 69 | Yes | No | Non-tongue | III | Moderate | 36 | 32 | 12.8 |
| P44 | Male | 72 | Yes | Yes | Non-tongue | III | Moderate | 36 | 36 | 13.8 |
| P45 | Male | 64 | No | No | Non-tongue | II | Moderate | 36 | 33 | 12 |
| P46 | Male | 68 | No | Yes | Non-tongue | III | Moderate | 13 | 10 | 15.5 |
| P47 | Male | 72 | Yes | Yes | Non-tongue | III | Well | 19 | 10 | 14.9 |
| P48 | Male | 66 | Yes | Yes | Non-tongue | III | Well | 12 | 10 | 14 |
| P49 | Female | 57 | No | Yes | Non-tongue | II | poor | 15 | 14 | 20 |
| P50 | Male | 70 | Yes | No | Tongue | III | Moderate | 36 | 36 | 15 |
| P51 | Male | 66 | No | Yes | Non-tongue | I | Moderate | 7 | 5 | 10.2 |
| P52 | Male | 72 | No | Yes | Non-tongue | IV | poor | 36 | 36 | 13.7 |
| P53 | Male | 68 | No | Yes | Tongue | II | Well | 36 | 36 | 12.5 |
| P54 | Female | 74 | Yes | No | Tongue | IV | Well | 36 | 36 | 13.2 |
| P55 | Female | 21 | No | Yes | Tongue | III | poor | 18 | 14 | 16.1 |
| P56 | Male | 63 | Yes | Yes | Tongue | I | poor | 36 | 32 | 15.9 |
| P57 | Male | 46 | Yes | No | Tongue | IV | Moderate | 36 | 36 | 10.7 |
| P58 | Male | 68 | Yes | No | Tongue | II | poor | 36 | 36 | 11.5 |
| P59 | Female | 35 | No | Yes | Non-tongue | IV | poor | 12 | 9 | 17.1 |
| P60 | Male | 26 | **No** | Yes | Non-tongue | II | Moderate | 36 | 36 | 20.2 |
| P61 | Male | 67 | No | Yes | Non-tongue | I | poor | 36 | 35 | 10.1 |
| P62 | Female | 71 | No | Yes | Tongue | II | Well | 30 | 21 | 10.9 |
| P63 | Female | 49 | No | Yes | Non-tongue | IV | Well | 36 | 36 | 11 |
| P64 | Male | 66 | Yes | Yes | Tongue | IV | Well | 36 | 36 | 15.9 |
| P65 | Male | 52 | **No** | Yes | Non-tongue | I | poor | 25 | 20 | 10.3 |
| P66 | Female | 74 | No | Yes | Tongue | III | Well | 36 | 36 | 10.6 |
| P67 | Male | 55 | No | Yes | Non-tongue | I | poor | 36 | 36 | 20 |
| P68 | Female | 61 | No | Yes | Tongue | I | Well | 36 | 36 | 18.3 |
| P69 | Female | 37 | Yes | No | Tongue | III | poor | 12 | 6 | 15.1 |
| P70 | Female | 27 | No | Yes | Non-tongue | IV | Moderate | 36 | 31 | 13.2 |
| P71 | Male | 65 | No | Yes | Tongue | I | poor | 36 | 36 | 13 |
| P72 | Female | 33 | Yes | Yes | Non-tongue | II | Moderate | 36 | 36 | 12.9 |
| P73 | Female | 66 | No | Yes | Non-tongue | III | poor | 17 | 15 | 19.1 |
| P74 | Female | 68 | Yes | Yes | Non-tongue | III | poor | 21 | 15 | 10.3 |
| P75 | Male | 66 | Yes | No | Non-tongue | II | Moderate | 27 | 25 | 10.9 |
| P76 | Male | 51 | Yes | No | Non-tongue | I | poor | 36 | 36 | 20.3 |
| P77 | Male | 68 | No | No | Non-tongue | III | Moderate | 36 | 36 | 19.8 |
| P78 | Male | 58 | **Yes** | No | Non-tongue | I | Moderate | 36 | 32 | 15.3 |
| P79 | Male | 60 | No | Yes | Tongue | IV | poor | 26 | 22 | 17.3 |
| P80 | Male | 34 | Yes | No | Tongue | III | Well | 30 | 24 | 12.8 |
| P81 | Male | 68 | **No** | Yes | Non-tongue | III | Moderate | 36 | 33 | 19.6 |
| P82 | Female | 42 | No | Yes | Non-tongue | II | poor | 13 | 10 | 18 |
| P83 | Male | 33 | Yes | No | Non-tongue | II | poor | 36 | 36 | 20.4 |
| P84 | Male | 68 | **No** | No | Non-tongue | IV | Well | 36 | 36 | 15.4 |
| P85 | Female | 39 | No | Yes | Non-tongue | II | poor | 36 | 36 | 19.5 |
| P86 | Female | 32 | Yes | No | Tongue | IV | poor | 20 | 16 | 17.1 |
| P87 | Female | 67 | No | Yes | Non-tongue | II | Well | 36 | 32 | 19.7 |
| P88 | Male | 57 | No | Yes | Non-tongue | I | Well | 36 | 36 | 11.9 |
| P89 | Male | 73 | Yes | Yes | Tongue | I | Well | 36 | 36 | 15 |
| P90 | Male | 63 | Yes | No | Tongue | I | Moderate | 36 | 34 | 11.3 |
| P91 | Female | 66 | Yes | No | Tongue | II | Well | 36 | 36 | 11.8 |
| P92 | Male | 63 | No | No | Tongue | III | Well | 23 | 14 | 17.2 |
| P93 | Female | 49 | Yes | Yes | Non-tongue | II | Moderate | 36 | 36 | 14.9 |
| P94 | Male | 67 | Yes | Yes | Tongue | II | poor | 18 | 15 | 16.9 |
| P95 | Male | 27 | Yes | No | Non-tongue | I | Moderate | 36 | 36 | 14.6 |
| P96 | Female | 71 | Yes | Yes | Tongue | I | poor | 36 | 36 | 10.6 |
| P97 | Female | 42 | No | Yes | Non-tongue | I | poor | 36 | 36 | 10.6 |
| P98 | Female | 63 | No | Yes | Tongue | II | Well | 36 | 36 | 13.7 |
| P99 | Male | 55 | Yes | Yes | Non-tongue | II | Well | 34 | 32 | 18 |
| P100 | Male | 73 | Yes | Yes | Tongue | I | Well | 36 | 36 | 14.9 |
| P101 | Male | 60 | Yes | Yes | Non-tongue | II | Well | 36 | 36 | 14.2 |
| P102 | Female | 40 | No | Yes | Non-tongue | I | Well | 36 | 36 | 10.4 |
| P103 | Male | 72 | Yes | No | Tongue | IV | Well | 35 | 29 | 20.8 |
| P104 | Female | 43 | Yes | Yes | Non-tongue | II | Moderate | 36 | 36 | 20.3 |
| P105 | Male | 21 | No | No | Tongue | II | poor | 36 | 30 | 10.8 |
| P106 | Male | 71 | No | Yes | Non-tongue | I | Moderate | 36 | 36 | 10.9 |
| P107 | Female | 40 | Yes | Yes | Non-tongue | IV | poor | 26 | 21 | 16.7 |
| P108 | Female | 25 | No | Yes | Non-tongue | III | Moderate | 10 | 8 | 19.6 |
| P109 | Female | 73 | **No** | Yes | Non-tongue | III | poor | 20 | 16 | 18.2 |
| P110 | Male | 73 | No | Yes | Tongue | IV | poor | 22 | 19 | 18.9 |
| P111 | Male | 43 | Yes | Yes | Non-tongue | I | Moderate | 36 | 36 | 16.1 |
| P112 | Male | 75 | Yes | Yes | Non-tongue | IV | poor | 36 | 36 | 19 |
| P113 | Male | 46 | No | No | Tongue | III | poor | 24 | 17 | 19.2 |
| P114 | Male | 40 | Yes | Yes | Non-tongue | IV | Moderate | 36 | 36 | 16 |
| P115 | Male | 38 | No | Yes | Non-tongue | IV | Well | 16 | 7 | 17.6 |
| P116 | Male | 42 | Yes | Yes | Non-tongue | III | Well | 36 | 36 | 19.5 |
| P117 | Male | 75 | No | Yes | Tongue | II | Moderate | 22 | 15 | 16.3 |
| P118 | Male | 71 | No | Yes | Non-tongue | II | Moderate | 36 | 36 | 14.3 |
| P119 | Female | 47 | No | Yes | Non-tongue | II | poor | 36 | 36 | 12.7 |
| P120 | Male | 65 | Yes | No | Tongue | III | Well | 34 | 30 | 20.1 |
| P121 | Male | 51 | Yes | No | Tongue | II | Well | 36 | 36 | 18.3 |
| P122 | Male | 75 | Yes | No | Non-tongue | I | Moderate | 36 | 36 | 14.1 |
| P123 | Male | 57 | No | Yes | Non-tongue | II | Well | 36 | 36 | 11.2 |
| P124 | Male | 73 | **No** | Yes | Non-tongue | I | Moderate | 36 | 36 | 12.9 |
| P125 | Male | 21 | Yes | Yes | Non-tongue | IV | poor | 28 | 25 | 11.6 |
| P126 | Male | 60 | Yes | Yes | Non-tongue | II | Moderate | 36 | 36 | 10.6 |
| P127 | Male | 51 | No | Yes | Tongue | II | Well | 36 | 36 | 12.4 |
| P128 | Male | 59 | Yes | No | Tongue | IV | poor | 32 | 22 | 17.5 |
| P129 | Female | 22 | No | Yes | Non-tongue | III | poor | 24 | 20 | 17.7 |
| P130 | Male | 43 | Yes | Yes | Non-tongue | I | Moderate | 31 | 29 | 16.5 |
| P131 | Male | 70 | Yes | Yes | Non-tongue | IV | Moderate | 36 | 36 | 19.6 |
| P132 | Female | 66 | Yes | No | Tongue | IV | Well | 36 | 36 | 10.7 |
| P133 | Male | 63 | Yes | Yes | Tongue | I | poor | 36 | 36 | 12.5 |
| P134 | Male | 70 | No | Yes | Tongue | I | poor | 25 | 19 | 20.2 |
| P135 | Female | 45 | **No** | Yes | Non-tongue | II | Well | 36 | 36 | 11 |
| P136 | Male | 75 | Yes | No | Tongue | III | poor | 36 | 36 | 19.4 |
| P137 | Female | 72 | Yes | Yes | Non-tongue | II | poor | 17 | 15 | 19.7 |
| P138 | Female | 72 | Yes | Yes | Tongue | III | Moderate | 36 | 36 | 15.1 |
| P139 | Female | 30 | No | Yes | Non-tongue | IV | poor | 36 | 33 | 11.8 |
| P140 | Male | 72 | Yes | Yes | Non-tongue | IV | poor | 13 | 7 | 16.5 |
| P141 | Male | 67 | No | Yes | Non-tongue | II | Well | 36 | 36 | 16.9 |
| P142 | Female | 43 | Yes | Yes | Non-tongue | IV | Moderate | 13 | 8 | 17.4 |
| P143 | Male | 69 | No | Yes | Tongue | II | Well | 36 | 36 | 11 |
| P144 | Female | 72 | No | Yes | Tongue | II | Moderate | 36 | 35 | 10.1 |
| P145 | Female | 23 | Yes | Yes | Non-tongue | III | Well | 36 | 36 | 13 |
| P146 | Female | 51 | Yes | Yes | Non-tongue | III | poor | 28 | 23 | 12.9 |
| P147 | Male | 29 | Yes | Yes | Non-tongue | II | Moderate | 36 | 36 | 18.1 |
| P148 | Male | 62 | No | Yes | Tongue | III | Well | 36 | 36 | 17.6 |
| P149 | Male | 73 | Yes | No | Non-tongue | IV | poor | 36 | 36 | 11.7 |
| P150 | Male | 74 | Yes | No | Non-tongue | I | Well | 27 | 19 | 18.1 |
| P151 | Male | 48 | Yes | No | Tongue | III | Moderate | 36 | 36 | 12.3 |
| P152 | Female | 52 | Yes | No | Tongue | I | Moderate | 36 | 36 | 15.8 |
| P153 | Male | 65 | Yes | No | Tongue | IV | Well | 36 | 36 | 17.2 |
| P154 | Female | 42 | Yes | Yes | Non-tongue | II | Moderate | 36 | 36 | 10.9 |
| P155 | Female | 62 | No | Yes | Non-tongue | II | Moderate | 36 | 32 | 18.3 |
| P156 | Male | 27 | Yes | Yes | Non-tongue | I | Moderate | 36 | 36 | 14.4 |
| P157 | Female | 73 | Yes | No | Non-tongue | II | Well | 9 | 6 | 10.6 |
| P158 | Male | 23 | Yes | No | Tongue | II | Moderate | 36 | 36 | 13 |
| P159 | Female | 34 | Yes | Yes | Tongue | IV | Well | 36 | 36 | 11.4 |
| P160 | Male | 72 | No | Yes | Non-tongue | III | Moderate | 19 | 17 | 20.8 |
| P161 | Male | 66 | Yes | Yes | Tongue | IV | Well | 36 | 36 | 20.5 |
| P162 | Male | 63 | Yes | Yes | Tongue | I | Moderate | 36 | 31 | 12.8 |
| P163 | Male | 59 | Yes | Yes | Tongue | I | Moderate | 36 | 36 | 12.8 |
| P164 | Male | 44 | Yes | Yes | Non-tongue | IV | poor | 20 | 19 | 13.7 |
| P165 | Male | 22 | No | Yes | Tongue | II | Moderate | 36 | 31 | 17.2 |
| P166 | Female | 73 | Yes | Yes | Non-tongue | IV | poor | 23 | 18 | 16.6 |
| P167 | Male | 75 | Yes | Yes | Non-tongue | III | Moderate | 36 | 34 | 18.1 |
| P168 | Male | 73 | Yes | Yes | Non-tongue | III | poor | 36 | 34 | 18.4 |
| P169 | Male | 71 | Yes | Yes | Non-tongue | I | Moderate | 36 | 36 | 14.4 |
| P170 | Male | 68 | No | Yes | Tongue | III | poor | 36 | 31 | 15.3 |
| P171 | Male | 55 | No | No | Tongue | I | Well | 16 | 14 | 13.2 |
| P172 | Female | 74 | Yes | Yes | Tongue | II | poor | 36 | 36 | 10.5 |
| P173 | Male | 47 | Yes | Yes | Tongue | III | Well | 16 | 13 | 18.5 |
| P174 | Male | 73 | No | No | Non-tongue | I | poor | 36 | 32 | 11.6 |
| P175 | Male | 30 | Yes | Yes | Non-tongue | III | poor | 5 | 4 | 19.3 |
| P176 | Female | 20 | Yes | No | Non-tongue | I | poor | 36 | 36 | 18.2 |
| P177 | Female | 68 | No | Yes | Tongue | I | Well | 36 | 36 | 15 |
| P178 | Male | 70 | No | Yes | Tongue | I | poor | 32 | 29 | 19.1 |
| P179 | Male | 62 | Yes | Yes | Tongue | III | Well | 36 | 36 | 14 |
| P180 | Female | 61 | Yes | No | Tongue | II | Well | 36 | 31 | 14.7 |
| P181 | Female | 71 | Yes | Yes | Tongue | II | poor | 36 | 36 | 18.8 |
| P182 | Male | 75 | No | Yes | Tongue | III | poor | 36 | 36 | 11.8 |
| P183 | Female | 62 | Yes | Yes | Tongue | II | Well | 36 | 36 | 17 |
| P184 | Female | 22 | No | Yes | Tongue | III | Moderate | 28 | 19 | 15.1 |

**Table 2.** **Clinical characteristics of controls**

| Control ID | age | gender | smoking | drinking | miR-130a值 |
| --- | --- | --- | --- | --- | --- |
| HC1 | 70 | Male | No | Yes | 11.1 |
| HC2 | 32 | Male | No | Yes | 9.8 |
| HC3 | 62 | Male | Yes | Yes | 12.5 |
| HC4 | 74 | Male | No | Yes | 7.8 |
| HC5 | 43 | Male | Yes | Yes | 13.8 |
| HC6 | 72 | Male | No | No | 13.8 |
| HC7 | 25 | Female | No | No | 6.4 |
| HC8 | 37 | Male | Yes | No | 6.8 |
| HC9 | 53 | Male | No | Yes | 7.1 |
| HC10 | 34 | Male | No | Yes | 12.1 |
| HC11 | 70 | Male | No | No | 16 |
| HC12 | 71 | Male | No | Yes | 14.8 |
| HC13 | 60 | Male | No | Yes | 9.6 |
| HC14 | 55 | Female | Yes | Yes | 7 |
| HC15 | 70 | Male | No | Yes | 7.4 |
| HC16 | 70 | Male | Yes | Yes | 8.8 |
| HC17 | 37 | Male | Yes | Yes | 7.1 |
| HC18 | 72 | Male | Yes | Yes | 8.5 |
| HC19 | 65 | Male | No | No | 15.5 |
| HC20 | 62 | Female | Yes | Yes | 15.6 |
| HC21 | 70 | Male | Yes | Yes | 6.9 |
| HC22 | 62 | Female | Yes | No | 9.9 |
| HC23 | 34 | Male | No | Yes | 11.2 |
| HC24 | 66 | Male | No | Yes | 7 |
| HC25 | 26 | Male | No | Yes | 12.3 |
| HC26 | 48 | Female | No | Yes | 7.2 |
| HC27 | 68 | Female | No | Yes | 9 |
| HC28 | 29 | Male | No | Yes | 7.1 |
| HC29 | 71 | Male | No | Yes | 6.1 |
| HC30 | 75 | Male | No | Yes | 12.3 |
| HC31 | 60 | Male | No | Yes | 10.8 |
| HC32 | 33 | Male | No | Yes | 6.9 |
| HC33 | 63 | Male | Yes | Yes | 7.7 |
| HC34 | 75 | Male | No | Yes | 7.1 |
| HC35 | 73 | Male | Yes | Yes | 13.7 |
| HC36 | 37 | Male | Yes | No | 8.8 |
| HC37 | 33 | Male | No | No | 11.3 |
| HC38 | 68 | Male | Yes | Yes | 9.6 |
| HC39 | 62 | Male | No | No | 6.4 |
| HC40 | 48 | Female | Yes | Yes | 12.1 |
| HC41 | 44 | Female | Yes | No | 7.7 |
| HC42 | 73 | Female | No | Yes | 7 |
| HC43 | 21 | Male | No | Yes | 6.8 |
| HC44 | 33 | Male | No | Yes | 12.2 |
| HC45 | 30 | Female | No | No | 6.1 |
| HC46 | 72 | Female | No | Yes | 12.4 |
| HC47 | 26 | Male | No | Yes | 7.7 |
| HC48 | 62 | Male | Yes | Yes | 13.6 |
| HC49 | 70 | Female | No | Yes | 6.6 |
| HC50 | 72 | Male | No | Yes | 6 |
| HC51 | 69 | Female | No | Yes | 13.6 |
| HC52 | 61 | Male | No | No | 11.1 |
| HC53 | 30 | Male | Yes | Yes | 13.5 |
| HC54 | 75 | Female | Yes | Yes | 15.7 |
| HC55 | 41 | Female | No | Yes | 8.6 |
| HC56 | 36 | Female | Yes | Yes | 11.9 |
| HC57 | 21 | Male | No | Yes | 10 |
| HC58 | 71 | Male | No | Yes | 8.8 |
| HC59 | 51 | Female | Yes | Yes | 15 |
| HC60 | 61 | Female | No | Yes | 8.5 |
| HC61 | 57 | Male | No | No | 10 |
| HC62 | 68 | Female | Yes | No | 8 |
| HC63 | 67 | Female | No | No | 8.7 |
| HC64 | 61 | Female | Yes | Yes | 13.5 |
| HC65 | 27 | Male | No | Yes | 10.6 |
| HC66 | 71 | Male | No | Yes | 9.6 |
| HC67 | 33 | Male | Yes | No | 8.8 |
| HC68 | 73 | Female | Yes | Yes | 10.9 |
| HC69 | 46 | Female | No | Yes | 11.5 |
| HC70 | 55 | Female | Yes | Yes | 8.7 |
| HC71 | 66 | Female | No | No | 13.1 |
| HC72 | 62 | Male | Yes | No | 10.3 |
| HC73 | 68 | Male | No | Yes | 10.7 |
| HC74 | 53 | Female | No | No | 9.8 |
| HC75 | 39 | Male | Yes | Yes | 14.2 |
| HC76 | 22 | Male | Yes | Yes | 15.2 |
| HC77 | 72 | Male | No | Yes | 16 |
| HC78 | 74 | Male | No | Yes | 12.5 |
| HC79 | 42 | Female | No | Yes | 13.6 |
| HC80 | 65 | Male | Yes | No | 10 |
| HC81 | 72 | Female | No | No | 15.8 |
| HC82 | 52 | Male | Yes | Yes | 10.9 |
| HC83 | 62 | Male | Yes | No | 8 |
| HC84 | 31 | Male | Yes | No | 11.8 |
| HC85 | 48 | Male | No | Yes | 13.9 |
| HC86 | 62 | Male | No | Yes | 8.6 |
| HC87 | 74 | Male | Yes | No | 11.7 |
| HC88 | 71 | Female | No | No | 12.8 |
| HC89 | 61 | Male | No | Yes | 6.5 |
| HC90 | 40 | Male | Yes | Yes | 14.1 |
| HC91 | 31 | Male | No | Yes | 15.1 |
| HC92 | 75 | Male | Yes | Yes | 13.2 |
| HC93 | 61 | Male | Yes | Yes | 7.4 |
| HC94 | 70 | Male | Yes | Yes | 9.8 |
| HC95 | 64 | Male | Yes | Yes | 12.8 |
| HC96 | 57 | Male | No | Yes | 15.5 |
| HC97 | 46 | Female | Yes | Yes | 6.7 |
| HC98 | 75 | Female | No | Yes | 12.6 |
| HC99 | 66 | Male | Yes | Yes | 10.9 |
| HC100 | 73 | Male | No | Yes | 13 |
| HC101 | 40 | Female | Yes | Yes | 7.7 |
| HC102 | 33 | Female | No | Yes | 15.6 |
| HC103 | 30 | Male | Yes | Yes | 7.8 |
| HC104 | 66 | Female | Yes | Yes | 14.7 |
| HC105 | 67 | Male | No | No | 8.2 |
| HC106 | 70 | Male | No | Yes | 16 |
| HC107 | 70 | Male | No | No | 15 |
| HC108 | 71 | Male | Yes | Yes | 9 |
| HC109 | 74 | Male | Yes | Yes | 7.1 |
| HC110 | 46 | Female | No | Yes | 10.6 |
| HC111 | 33 | Male | No | No | 14.7 |
| HC112 | 32 | Male | No | Yes | 14.8 |
| HC113 | 31 | Female | No | Yes | 11.8 |
| HC114 | 26 | Male | Yes | Yes | 9.9 |
| HC115 | 31 | Male | No | Yes | 10.9 |
| HC116 | 53 | Male | No | Yes | 13.2 |
| HC117 | 49 | Male | No | Yes | 14.5 |
| HC118 | 38 | Female | Yes | Yes | 10.7 |
| HC119 | 39 | Female | No | No | 9.8 |
| HC120 | 62 | Male | Yes | No | 15.7 |
| HC121 | 71 | Female | Yes | Yes | 15.8 |
| HC122 | 74 | Male | No | No | 10 |
| HC123 | 71 | Male | No | Yes | 13.7 |
| HC124 | 74 | Female | No | Yes | 15.4 |
| HC125 | 72 | Female | Yes | Yes | 15.4 |
| HC126 | 75 | Female | Yes | Yes | 15.9 |
| HC127 | 66 | Male | No | Yes | 11.2 |
| HC128 | 49 | Female | Yes | No | 8.8 |
| HC129 | 48 | Male | No | Yes | 7.4 |
| HC130 | 39 | Male | No | Yes | 13.5 |
| HC131 | 25 | Male | Yes | Yes | 9.6 |
| HC132 | 65 | Female | Yes | No | 6.2 |
| HC133 | 69 | Female | No | Yes | 13.2 |
| HC134 | 72 | Female | No | Yes | 12.7 |
| HC135 | 64 | Female | No | No | 8.2 |
| HC136 | 31 | Female | No | No | 14.9 |
| HC137 | 58 | Male | Yes | Yes | 6.4 |
| HC138 | 75 | Male | Yes | No | 10.3 |
| HC139 | 65 | Male | No | Yes | 10.7 |
| HC140 | 59 | Male | No | Yes | 13.9 |
| HC141 | 28 | Female | Yes | No | 14.6 |
| HC142 | 54 | Female | No | No | 13.1 |
| HC143 | 33 | Male | No | Yes | 6.3 |
| HC144 | 21 | Female | Yes | Yes | 9.6 |
| HC145 | 69 | Male | Yes | No | 11.2 |
| HC146 | 70 | Male | No | Yes | 13.3 |
| HC147 | 74 | Male | Yes | Yes | 12.8 |
| HC148 | 73 | Male | Yes | Yes | 9.6 |
| HC149 | 75 | Male | No | No | 13.3 |
| HC150 | 51 | Male | No | Yes | 13.5 |
| HC151 | 39 | Male | Yes | Yes | 8.9 |
| HC152 | 31 | Male | Yes | Yes | 10.1 |
| HC153 | 37 | Male | No | Yes | 7.9 |
| HC154 | 71 | Male | Yes | Yes | 11.3 |
| HC155 | 63 | Male | No | Yes | 10.3 |
| HC156 | 74 | Male | Yes | No | 14.6 |
| HC157 | 66 | Male | No | Yes | 11.7 |
| HC158 | 30 | Female | No | Yes | 11.7 |
| HC159 | 27 | Female | Yes | No | 12.6 |
| HC160 | 54 | Male | No | Yes | 12 |
| HC161 | 27 | Male | Yes | Yes | 15.2 |
| HC162 | 39 | Male | No | Yes | 9.2 |
| HC163 | 61 | Male | Yes | Yes | 15 |
| HC164 | 66 | Female | No | No | 12 |
| HC165 | 64 | Female | Yes | No | 13.2 |
| HC166 | 63 | Male | No | Yes | 13.4 |
| HC167 | 68 | Male | Yes | Yes | 14.3 |
| HC168 | 51 | Male | Yes | Yes | 11.1 |
| HC169 | 39 | Female | Yes | Yes | 9 |
| HC170 | 47 | Female | Yes | Yes | 13.7 |
| HC171 | 25 | Male | No | Yes | 11.4 |
| HC172 | 24 | Male | No | Yes | 6.4 |
| HC173 | 38 | Female | No | Yes | 7.3 |
| HC174 | 62 | Male | No | Yes | 6.5 |
| HC175 | 67 | Male | Yes | Yes | 6.4 |
| HC176 | 58 | Female | No | Yes | 7.1 |
| HC177 | 47 | Female | Yes | Yes | 13.2 |
| HC178 | 66 | Female | Yes | Yes | 14.9 |
| HC179 | 75 | Male | No | Yes | 8.8 |
| HC180 | 72 | Male | Yes | No | 11.8 |
| HC181 | 68 | Male | Yes | Yes | 10.4 |
| HC182 | 65 | Female | Yes | No | 7.3 |
| HC183 | 35 | Male | Yes | No | 7.6 |
| HC184 | 48 | Male | No | Yes | 8.1 |
| HC185 | 72 | Male | Yes | Yes | 12.8 |
| HC186 | 66 | Male | Yes | Yes | 7.8 |
| HC187 | 75 | Male | Yes | Yes | 12.4 |
| HC188 | 66 | Male | Yes | Yes | 8.9 |
| HC189 | 67 | Male | Yes | Yes | 12.8 |
| HC190 | 61 | Male | Yes | Yes | 14.6 |
| HC191 | 72 | Male | Yes | Yes | 13.9 |
| HC192 | 71 | Male | Yes | Yes | 10.7 |
| HC193 | 66 | Male | Yes | Yes | 6.4 |
| HC194 | 66 | Male | Yes | Yes | 7 |
| HC195 | 68 | Male | Yes | Yes | 10.2 |
| HC196 | 66 | Male | Yes | Yes | 11.6 |
